# Time-course microarray transcriptome data of *in vitro* cultured testes and age-matched *in vivo* testes

**DOI:** 10.1016/j.dib.2020.106482

**Published:** 2020-10-31

**Authors:** Takeru Abe, Hajime Nishimura, Takuya Sato, Harukazu Suzuki, Takehiko Ogawa, Takahiro Suzuki

**Affiliations:** 1 Biopharmaceutical and Regenerative Sciences, Graduate School of Medical Life Science, Yokohama City University, Yokohama, Kanagawa, Japan; 2 Laboratory for Cellular Function Conversion Technology, RIKEN Center for Integrative Medical Sciences, Yokohama, Kanagawa, Japan; 3 Functional Genomics, Graduate School of Medical Life Science, Yokohama City University, Yokohama, Kanagawa, Japan

**Keywords:** Spermatogenesis, Organ culture, Transcriptome, Microarray

## Abstract

*In vitro* spermatogenesis, which produces fertile spermatozoa, has been successfully performed using an organ culture method from murine tissue. Here, we provide a dataset of time-course microarray transcriptome data of *in vitro* cultured neonate murine testes and age-matched *in vivo*-derived testes. The dataset presented here is related to the article titled “Transcriptome analysis reveals inadequate spermatogenesis and immediate radical immune reactions during organ culture in vitro spermatogenesis” published in Biochemical and Biophysical Research Communications in 2020 [1]. The raw data and pre-processed data are publicly available on the GEO repository (accession number GSE147982). Furthermore, the dataset provided here includes additional metadata, detailed explanations of the experiment, results of pre-processing, analysis scripts, and lists of differentially expressed genes from *in vitro* culture testes and *in vivo* testes at each time point.

## Specifications Table

**Table:** 

Subject	Molecular Biology
Specific subject area	Andrology
Type of data	Binary Table Figure
How data were acquired	Illumina MouseWG-6 v2.0 Expression beadchip
Data format	Raw Analyzed
Parameters for data collection	*In vitro* cultured testes for 2, 4, 6, 7, 9, and 14 days from 7 day-post-partum (dpp) mice and time-matched *in vivo-*derived testes of corresponding age of mice to the cultured testes (7, 9, 11, 13, 14, 16, and 21 dpp).
Description of data collection	Mouse testes were extracted from 7 dpp male *Acr-Gfp*^+/+^ or *Acr-Gfp*^+/−^ mice (a mixture of ICR and C57BL/6) and cultured by the gas-liquid interphase culture method. The *in vitro* cultured testes are then collected and subjected to total RNA extraction. The total RNA from *in vivo*-derived testes of corresponding aged mice to the cultured testes were also extracted. The quality of the total RNA was checked and microarray analysis were performed by Illumina MouseWG-6 v2.0 Expression beadchip.
Data source location	RIKEN Center for Integrative Medical Sciences 1-7-22 Suehiro-cho, Tsurumi-ku, Yokohama, Kanagawa, Japan
Data accessibility	Repository name: Gene Expression Omnibus Data identification number: GSE147982 Direct URL to data: https://www.ncbi.nlm.nih.gov/geo/query/acc.cgi?acc=GSE147982
Related research article	Takeru Abe, Hajime Nishimura, Takuya Sato, Harukazu Suzuki, Takehiko Ogawa, Takahiro Suzuki, Time-course microarray transcriptome data of in vitro cultured testes and corresponding in vivo testes. *Biochem Biophys Res Commun.* 530 (2020) 732-738 https://doi.org/10.1016/j.bbrc.2020.06.161

## Value of the Data

• This is the first time-course transcriptome data of *in vitro* cultured testes, which is useful for describing the difference between *in vitro* cultured testes and *in vivo* testes.
• The data benefits scientists in reproductive medicine and reproductive engineering, especially those involved in *in vitro* spermatogenesis.
• The data can be used to identify target pathways/genes to improve *in vitro* spermatogenesis.

## 1 Data Description

**Fig. 1:**
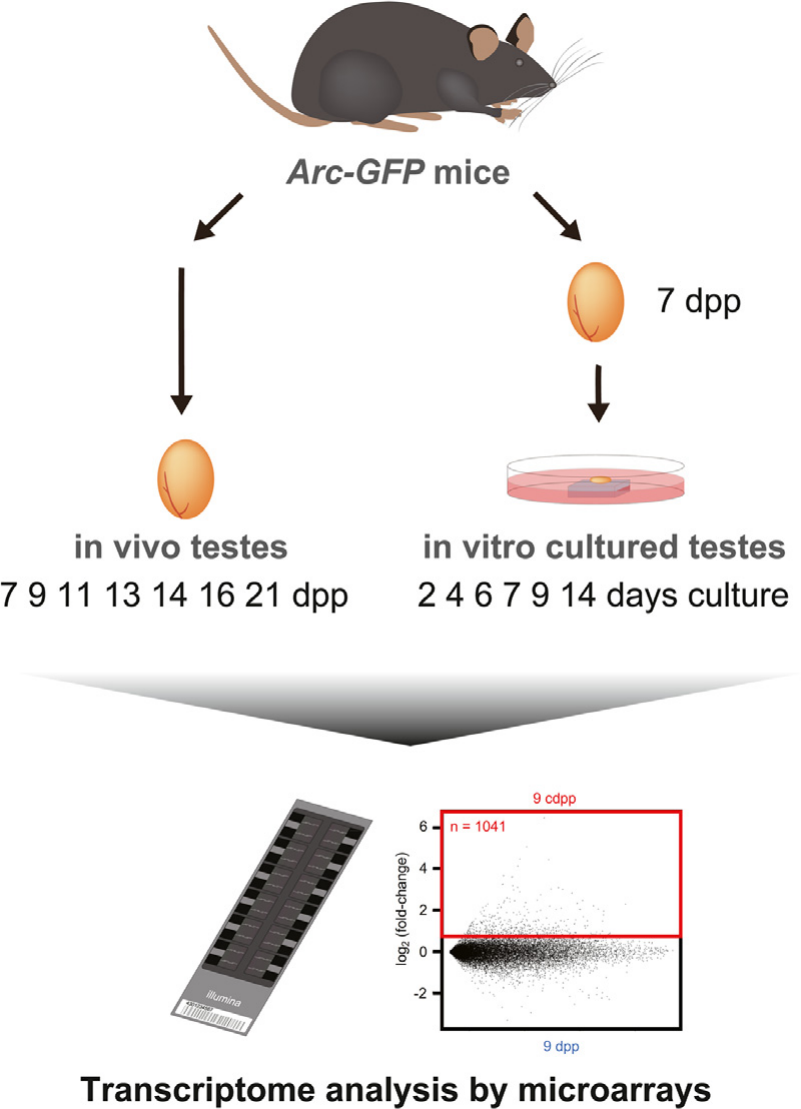
Schematic representation of data generation.

**Fig. 2:**
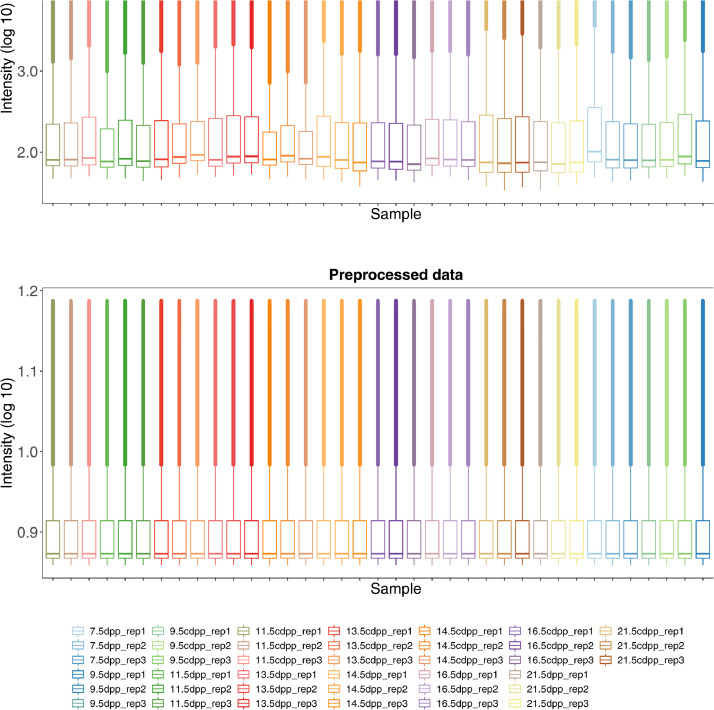
Distribution of signal intensity. The log_10_ transformed signal intensity of each sample is shown as boxplot. The upper panel is raw intensity (before pre-processing) and the lower panel is after pre-processing. The abbreviation “dpp” corresponds days post-partum (days-old) and “cddp” corresponds corresponding days post-partum, which is age-matched sample to the *in vivo* derived testes. Rep means biological replicate index of an experiment.

RNA was extracted from *in vitro* cultured testes (2, 4, 6, 7, 9, and 14 days of culture from 7 dpp mouse testes) and age-matched control *in vivo*-derived samples (7, 9, 11, 13, 14, 16, and 21 dpp mouse testes), followed by microarray analyses in three biological replicates. A schematic representation of the data generation is shown in Fig. 1. The raw intensity binary and text data of Illumina MouseWG-6 v2.0 Expression beadchip are provided as supplementary files (GSE147982_RAW.tar and GSE147982_non-normalized.txt.gz files, respectively) at Gene Expression Omnibus (GEO) (https://www.ncbi.nlm.nih.gov/geo/) with the accession number GSE147982. The link between each GEO sample accession and the original mouse is provided in Table 1. The preprocessed data, which is subjected to background correction, variance-stabilizing transformation (VST), and quantile normalization, are also provided at GEO GSE147982. The distributions of signal intensity of raw and preprocessing data are reported in Fig. 2. Supplementary Table 1 provides the result of differential expression analysis, which is done using an empirical moderated t-statistics test with a cut-off adjusted p-value of 0.05.

**Table 1:** Origin of RNA sample. The abbreviation “dpp” corresponds days post-partum (days-old) and “cddp” corresponds corresponding days post-partum, which is age-matched sample to the *in vivo* derived testes. Rep means biological replicate index of an experiment.

Mouse ID	Weight (g)	GEO sample accession		
1	3.66	GSM4451319		
2	5.33	GSM4451321		
3	4.570	GSM4451323		
4	5.54	GSM4451331		
5	4.37	GSM4451332		
6	5.71	GSM4451333		
7	7.13	GSM4451334		
8	6.15	GSM4451335		
9	5.99	GSM4451336		
10	6.73	GSM4451337		
11	5.63	GSM4451338		
12	6.43	GSM4451339		
13	8.78	GSM4451320		
14	7.07	GSM4451322		
15	7.68	GSM4451324		
16	7.85	GSM4451340		
17	6.46	GSM4451341		
18	6.05	GSM4451342		
19	18.36	GSM4451325		
20	17.51	GSM4451326		
21	14.14	GSM4451327		
22	4.31	GSM4451343		
23	5.88	GSM4451344		
24	5.19	GSM4451345		
25	4.31	GSM4451346	GSM4451349	GSM4451352
26	5.88	GSM4451347	GSM4451350	GSM4451353
27	5.19	GSM4451348	GSM4451351	GSM4451354
28	4.14	GSM4451355	GSM4451328	
29	5.5	GSM4451356	GSM4451329	
30	5.28	GSM4451357	GSM4451330

## 2 Experimental Design, Materials and Methods

### 2.1 Mice

*Acr-Gfp* transgenic mice (C57BL/6 strain) [2] were obtained from RIKEN BRC through the National Bio-Resource Project of MEXT, Japan. Male homozygous *Acr-Gfp* transgenic mice were bred with female ICR, C57BL/6 (CLEA Japan, Tokyo, Japan), or ICRxC57BL/6 F1. Thus, male mice of *Acr-Gfp* (+/+) or (+/-) background were used for analysis. Mice were housed in the TSRI Specific Pathogen Free (SPF) facility with a 14-hour light cycle at 24 ± 1°C and 55 ± 5% air conditions and were given hard pellet food (Oriental Yeast, Tokyo, Japan) and acidified water (pH 2.8–3.0) ad libitum.

### 2.2 Organ culture

Testes were extracted from 7 dpp male *Acr-Gfp* transgenic mice. The extracted testes were decapsulated and cut into 4-8 fragments by forceps under a microscope in the culture medium (α-minimum essential medium (Thermo Fisher Scientific Inc., Wilmington, NC, USA)), supplemented with 40 mg/mL AlbuMAX (Thermo Fisher Scientific Inc.). The testis fragments were then patterned onto a 1.5% (w/v) agarose gel block (about 10 mmW × 10 mmD × 5 mmH) that was pre-soaked for at least two days in advance and then half-submerged in the culture medium in a well of a 12 well tissue-culture plate (Greiner Bio-One, Kremsmünster, Austria). The testes fragments were covered with the Polydimethylsiloxane (PDMS)-ceiling chip [3] and cultured in 5% CO_2_ at 34°C with once a week medium replacement.

### 2.3 RNA extraction

Fresh *in vivo*-derived testes were maintained in RNA later (Thermo Fisher Scientific Inc.) on ice for at least 3 h until RNA extraction. The *in vivo-*derived testes and *in vitro* cultured testes were homogenized in TRIzol Reagent (Thermo Fisher Scientific Inc.) with the PT1300D polytron homogenizer (KINEMATICA AG, Luzern, Switzerland), and the aqueous phase was collected according to the manufacturer's instructions. RNA was extracted from the corrected aqueous phase using the NucleoSpin RNA (MACHEREY-NAGEL, Düren, Germany), according to the manufacturer's instructions. The quality of the RNA was confirmed by NanoDrop (ND-1000, Thermo Fisher Scientific Inc.) and RNA 6000 nano kit of Bioanalyzer (Agilent Technologies, Santa Clara, CA, USA). RNA samples with high RNA integrity values (> 9) were used in the microarray analyses.

### 2.4 Microarray

Total RNA was amplified by *in vitro* transcription method and the resulting cRNAs were biotinylated using the Illumina TotalPrep RNA Amplification Kit (Illumina, Inc., San Diego, CA, USA). The biotinylated cRNA was hybridized to MouseWG-6 v2.0 Expression beadchip (Illumina, Inc.). Signal intensity was measured using the Illumina BeadArray reader (Illumina, Inc.).

### 2.5 Data pre-processing

Raw intensity binary data (idat files) were converted to text data using BeadStudio (I llumina, Inc.) without background correction and normalization. Raw intensity text data were subjected to background correction, variance-stabilizing transformation (VST), and quantile normalization using *lumiExpresso* function implemented in the *lumi* package of R.

### 2.6 Differential expression analysis

The probes whose detection p-value was greater than 0.01 in any of the samples were removed. A linear model was fitted to the expression data for each probe using the *lmFit* function implemented in the *limma* package of R. Then, p-values of empirical Bayes moderated t-statistics test between *in vitro* cultured- and *in vivo*-derived-testes at each time point were computed by *contrasts.fit* and *eBayes* functions implemented in the *limma* package of R, followed by p-values adjustment by Benjamin*-*Hochberg method. The probes were considered to be differentially expressed genes if their adjusted p-value was less than 0.05.

### 2.7 Scripts

Scripts and data for R analysis used in this paper are available on GitHub (https://github.com/RIKEN-CFCT/ivs_data_in_brief).

## Declaration of Competing Interest

The authors have no conflicts of interest to declare.

## Appendix Supplementary materials

Image, application 1
